# Distinct pathological changes of osteochondral units in early OVX-OA involving TGF-β signaling

**DOI:** 10.3389/fendo.2022.1074176

**Published:** 2022-12-15

**Authors:** Zihuan Yang, Qizhao Tan, Zhenda Zhao, Guodong Niu, Siwei Li, Weishi Li, Chunli Song, Huijie Leng

**Affiliations:** ^1^ Department of Orthopedics, Peking University Third Hospital, Beijing, China; ^2^ Department of Orthopedics, Ansteel Group Hospital, Anshan, China; ^3^ Engineering Research Center of Bone and Joint Precision Medicine, Beijing, China; ^4^ Beijing Key Laboratory of Spinal Disease Research, Beijing Municipal Science & Technology Commission, Beijing, China

**Keywords:** osteoarthritis, estrogen deficiency, cartilage, subchondral bone, TGF-β signaling

## Abstract

**Introduction:**

Different opinions exist about the role of subchondral bone in osteoarthritis (OA), probably because subchondral bone has different effects on cartilage degeneration in OA induced by different pathologies. Animal studies to illustrate the role of subchondral bone in cartilage degeneration were mostly based on post-traumatic OA (PT-OA). Postmenopausal women experience a much higher occurrence of OA than similar-aged men. The physiological changes and pathogenesis of the osteochondral unit in ovariectomy-induced OA (OVX-OA) might be distinct from other types of OA.

**Methods:**

The osteochondral alterations of post-traumatic OA (PT-OA) and OVX-OA at week 9 after surgery were compared. Then the alterations of osteochondral units in OVX-OA rats were tracked over time for the designed groups: Sham, OVX and OVX rats treated with estrogen (OVX+E). DXA, micro-CT, and histochemical staining were performed to observe alterations in osteochondral units.

**Results:**

Rapid cartilage degeneration and increased bone formation were observed in PT-OA, while only mild cartilage erosion and significant bone loss were observed in OVX-OA at week 9 after surgery. Subchondral bone degradation preceded cartilage degeneration by 6 weeks in OVX-OA. TGF-β expression was downregulated in the osteochondral unit of OVX rats. Estrogen supplementation inhibited subchondral bone loss, cartilage degradation and TGF-β expression decrease.

**Discussion:**

This research demonstrated the distinct behaviors of the osteochondral unit and the critical role of subchondral bone in early OVX-OA compared with PT-OA. Inhibiting subchondral bone catabolism at the early stage of OVX-OA could be an effective treatment for post-menopausal OA. Based on the results, estrogen supplementation and TGF-β modulation at the early stage are both potential therapies for post-menopausal OA.

## 1 Introduction

Osteoarthritis (OA) is one of the most prevalent joint diseases and affects millions of people worldwide, especially elderly women (1). OA involves degeneration of the entire joint rather than simple articular cartilage damage (2). The close anatomic association between subchondral bone and articular cartilage, called the osteochondral unit, enables them to interact in both healthy and osteoarthritic joints (3).

Subchondral bone exhibits a diversity of changes and plays a variety of roles in OA induced by different pathologies (4, 5). Most current animal studies investigating the role of subchondral bone in cartilage degeneration are based on post-traumatic OA (PT-OA) (6). Menopausal women account for most of the population with OA (7). The ovariectomy-induced OA (OVX-OA) model, the most appropriate model to imitate postmenopausal OA (8), displays severe osteoporosis in subchondral bone and mild cartilage degeneration at week 9 after surgery (9, 10). Subchondral bone showed a high possibility of initiating and promoting cartilage degradation in OVX-OA. To date, there have been no reports on the evolution of changes in cartilage and subchondral bone over time at the early stage of OVX-OA, which will be helpful to better understand the process of postmenopausal OA.

Transforming growth factor-beta (TGF-β) is a homeostasis regulator for both subchondral bone and articular cartilage (11, 12). Cao’s group found that TGF-β activation is responsible for abnormal subchondral bone formation in anterior cruciate ligament transaction (ACLT)-induced OA (11). Lv’s group proved that TGF-β regulates age-related degeneration of the osteochondral unit in a spontaneous OA model (13). Estrogen can modulate TGF-β expression in various cells, including chondrocytes, osteoblasts and osteoclasts (14, 15). The present study aims to reveal the distinct features of OVX-OA and the correlation of the two constituents of the osteochondral unit over time under estrogen deficiency. We hypothesize that subchondral bone changes precede cartilage degeneration involving TGF-β signaling at the early stage of OVX-OA.

## 2 Materials and methods

### 2.1 Animal and treatments

All experimental operations were in accordance with the standard of Guidelines for the Care and Use of Laboratory Animals and were approved by the Animal Ethics Committee of Peking University (ethics approved number: LA2019209). Ninety-one six-month-old female Sprague–Dawley (SD) rats were purchased and raised in the Peking University Health Science Center with the controlled temperature (23°C ~ 25°C), humidity (40% ~ 50%), 12 h light/dark cycle and free access to food and water.

The rats were randomly divided into four groups as follows (1): Sham group (n = 28) (2); OVX group (n = 28) (3); PT-OA group (underwent ACLT, n = 7); and (4) OVX+E group (treated with estrogen, n = 28). The groups of Sham, OVX and OVX+E had more rats to investigate changes in the osteochondral units in OVX-OA over time (at week 3, 6, 9, and 18 after surgery, n = 7 for each time point). Three days after surgery, estrogen (100 μg/kg/d, once per day) was injected subcutaneously into rats in the OVX+E group. The body weights of all the rats were measured regularly. The 7 rats in the PT-OA group were sacrificed at week 9 after surgery. At week 3, 6, 9, and 18 after surgery, seven rats from the other groups were randomly selected and sacrificed. The knee joints and uterus were excised. The right knee joint of each rat was used for DXA and micro-CT analysis. The left knee joint of each rat was immediately fixed in 4% paraformaldehyde.

### 2.2 BMD measurements

To evaluate the bone mass changes of subchondral bone, the bone mineral density (BMD) was measured using the rat mode of dual-energy X-ray absorptiometry (DXA) (Discovery™, Hologic Inc, Boston, MA, USA) at week 3, 6, 9, and 18 after surgery. The area from the epiphyseal line to the subchondral cortical bone of the tibia plateau was identified as the region of interest (ROI). The system was calibrated with standard phantoms.

### 2.3 Micro-CT analysis

The left knee joints were then scanned using micro-CT (Inveon, Siemens Medical Solutions, IL, USA) with the following sets: 13-μm isotropic voxel, 80-kV voltage, 500-μA current, 300-ms settling time, 360 projections per 360° and 200-ms exposure time. The obtained raw data were reconstructed using Inveon Acquisition Workplace (Version 5.0, Siemens Medical Solutions USA, Inc., IL, USA). For subchondral trabecular bone analysis, covering the whole subchondral bone in tibial plateaus was selected as the ROI. Bone volume/tissue volume (BV/TV), trabecular number (Tb. N), trabecular thickness (Tb. Th) and trabecular separation (Tb. Sp) were calculated using Inveon Research Workplace (Version 5.0, Siemens Medical Solutions, IL, USA).

### 2.4 Tissue fixation and histological processing

The left knee joints were fixed in 4% paraformaldehyde for 48 h and washed in PBS. After fixation, the specimens were decalcified in 10% ethylenediaminetetraacetic acid (EDTA; pH= 7.4) for 6 weeks. Then, the specimens were embedded, sliced (5 µm) and processed for toluidine blue staining, immunohistochemistry staining and TRAP staining following the corresponding protocols (16, 17).

### 2.5 Histological analysis of the osteochondral unit

Joint sections were stained with toluidine blue. Using the modified Mankin scoring system, the difference in osteochondral alterations was compared between the OVX-OA and PT-OA rats. The modified Mankin scoring system assessed four categories of OA features: articular structure, cellularity, cartilage staining, and tidemark (18). No significant differences were detected between the Sham and OVX-OA groups based on the Mankin scoring, probably due to its insensitivity to OVX-induced early cartilage degeneration. Thus, when investigating osteochondral unit changes over time in OVX-OA, erosion severity was adopted to evaluate cartilage degeneration, as suggested in the literature (8). The length of the erosion surface (LES) and the length of the total cartilage surface (LTS) were measured using Image-Pro Plus (Version 6.0, Media Cybernetics, USA). The ratio of LES to LTS was defined as the severity score (8).

### 2.6 Immunostaining

Slices were dewaxed in xylene and rehydrated in ethanol grading. After rehydration, slices were quenched in 0.3% hydrogen peroxide in methanol. The rehydrated sections were subjected to antigen retrieval in gastric enzyme for 30 minutes followed by incubation with primary antibodies. The slides were incubated at 4 ° overnight with rabbit antibodies against TGF-β (Abcam, Cambridge, MA), type II collagen (Abcam, Cambridge, MA), Smad2/3 (Abcam, Cambridge, MA), Smad1/5/8 (Novus Biologicals, Inc., USA) at dilutions of 1:100, 1:100, 1:500 and 1:400 respectively, and then incubated with secondary antibodies (ZSGB-BIO, Beijing, China) at room temperature for 30 min. DAB (ZSGB-BIO, Beijing, China) was used to visualize the color. Images were captured by Image-Pro Plus using a digital slide scanner (Hamamatsu, Japan). The mean integrated optical density (IOD) was used to present the positive intensity of immunohistochemical expression. Three sections per sample were analyzed.

### 2.7 ELISA

To measure the protein levels of C-Telopeptides of Type I (CTX-I), Osteocalcin (OC), Matrix Metalloprotein-13 (MMP-13), Cartilage Oligomeric Matrix Protein (COMP) in rat serum, blood was collected after surgery at week 1, 2, 3, 5, 7, and 9. Serum was acquired by centrifugation at 4°C and 1500 r/min for 15 min. The serum was stored at -80°C until assayed. The expressions of CTX-I, OC, MMP-13 and COMP were measured by enzyme-linked immunosorbent assay (ELISA) using rat ELISA kits (Jiangsu Meimian industrial Co., Ltd, Yancheng, China; Immunoway Inc., TX, USA), in accordance with the manufacturer’s instructions.

### 2.8 Statistical analysis

All data are presented as the mean ± standard deviation (SD). Statistical analysis was carried out by GraphPad Prism 9.1.1 (GraphPad Software, Inc., CA, USA). Normal distribution was checked and confirmed before using an analysis of variance (ANOVA) with Tukey’s *post-hoc* test. A comparison of two groups was carried out by Student’s *t* test. Comparisons of at least three groups were evaluated by one-way ANOVA. Correlation between subchondral bone loss (BV/TV, Tb. N, Tb. Sp, Tb. Th) and cartilage erosion (Severity Score) was analyzed using Pearson correlation analysis. Values of p< 0.05 were regarded as statistically significant.

## 3 Results

### 3.1 The difference in osteochondral alterations between OVX-OA and PT-OA rats

**Figure 1:**
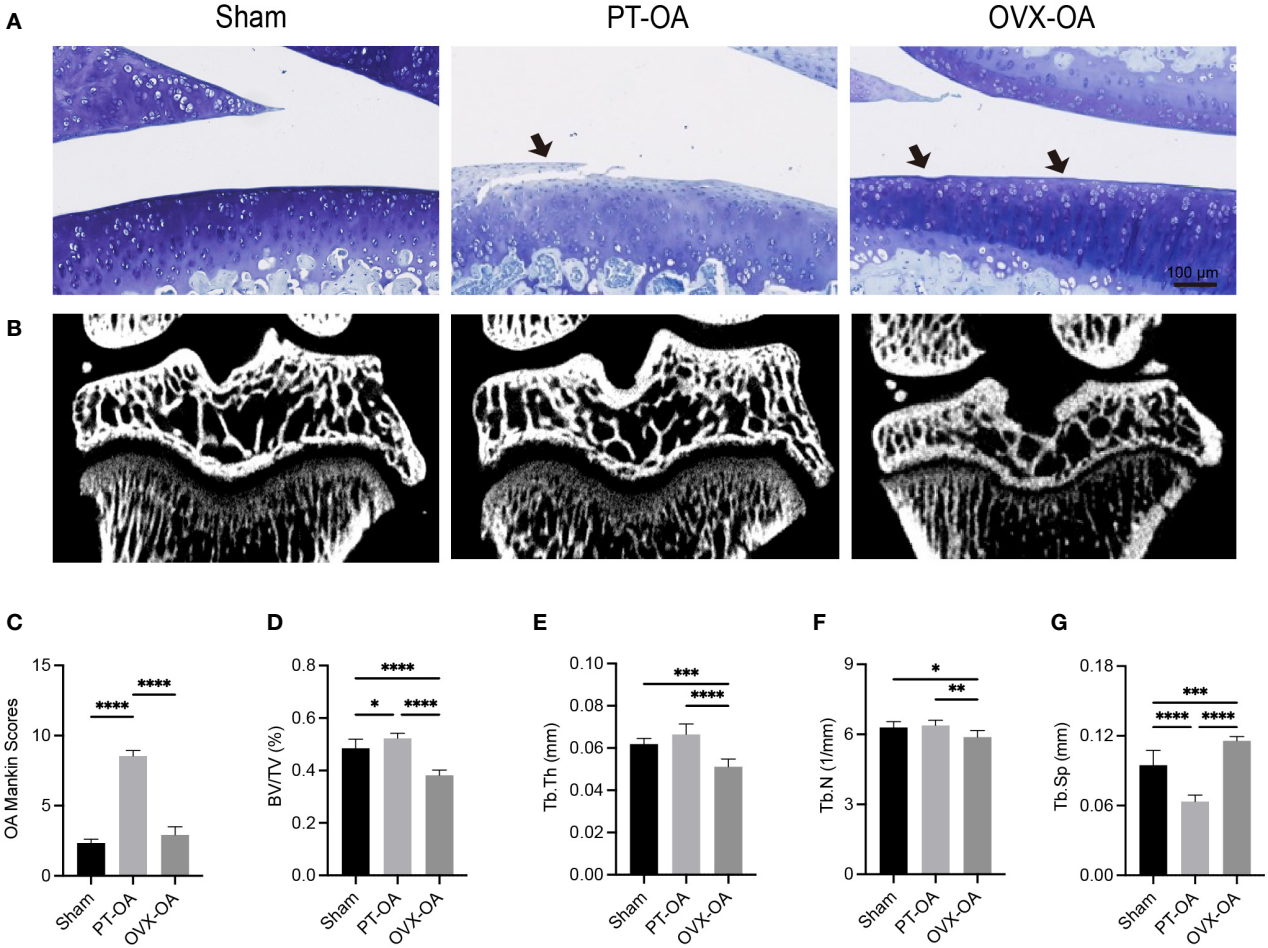
Comparison of subchondral bone and cartilage degeneration among Sham, PT-OA and OVX-OA. **(A)** Representative images of toluidine blue staining in cartilage (n = 7). Scale bar, 100 μm. **(B)** Representative μCT images of knee joints. **(C)** Mankin scores of knee joints. **(D–G)** Trabecular parameters of the subchondral bone (n = 7). *p< 0.05, **p< 0.01, ***p< 0.001, ****p< 0.0001. BV/TV, bone volume/tissue volume. Tb. Th, trabecular thickness. Tb. N, trabecular number. Tb. Sp, trabecular separation.

The osteochondral units of rats sacrificed at week 9 after surgery from Sham, PT-OA and OVX-OA groups were compared showing the distinct role of subchondral bone in OVX-OA (**Figure 1**). Cartilage defects and fissures in the middle and deep zones were observed in PT-OA rats. Only proteoglycan loss of cartilage and a mild rough surface were observed in OVX-OA rats (**Figure 1A**). The overall Mankin scores at week 9 after surgery in OVX-OA rats were significantly lower than those in PT-OA rats. The Mankin scoring system was not sensitive enough to detect significant differences in cartilage degeneration between the OVX and Sham groups (**Figure 1C**). Micro-CT images revealed that OVX rats experienced markedly higher subchondral bone loss than both Sham rats and PT-OA rats (**Figure 1B**). However, instead of bone loss, subchondral bone formation increased in PT-OA rats compared with OVX-OA rats, as shown by the significantly higher BV/TV, Tb. Th and Tb. N and lower Tb. Sp (**Figures 1D–G**).

### 3.2 Postoperative changes in subchondral bone of OVX rats over time

**Figure 2:**
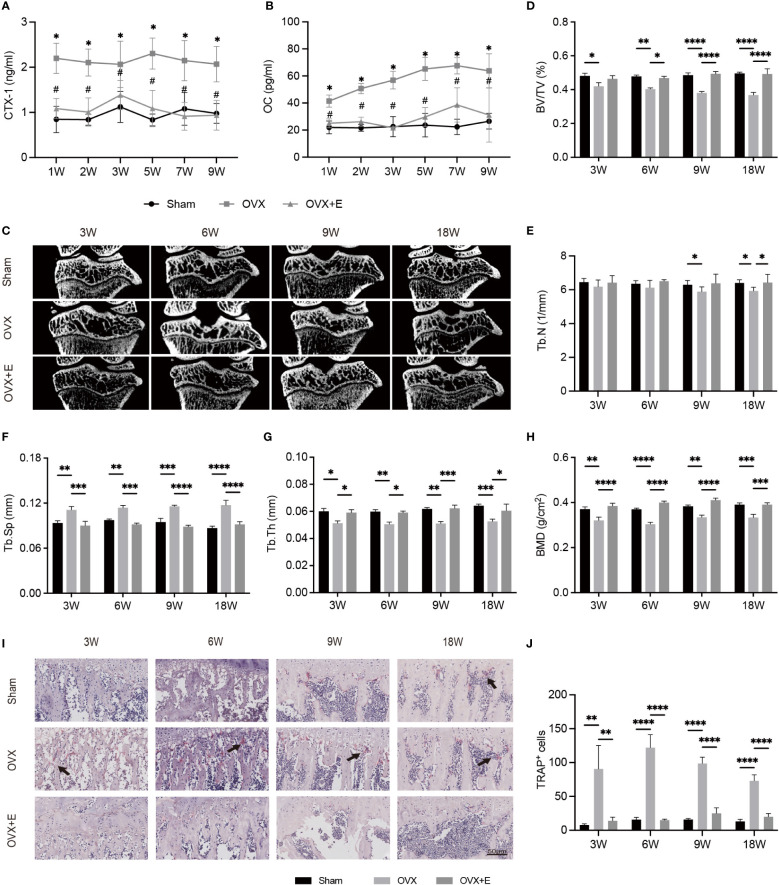
The alterations of subchondral bone after surgery from week 3 to week 18 in Sham, OVX and OVX+E rats. **(A, B)** The serum levels of CTX-I and OC (n = 7). *p< 0.05 vs. Sham group. #p< 0.05 vs. OVX group. **(C)** Representative μCT images of the proximal tibia (n = 7). **(D–G)** S ubchondral trabecular parameters (n = 7). **(H)** BMD (n = 7). **(I)** TRAP staining of the subchondral bone in the tibia. The black arrow indicates the activated osteoclasts (n = 7). **(J)** Quantitative analysis of the area of TRAP^+^ cells per bone marrow area (mm^2^). Scale bar, 50 μm. *p< 0.05, **p< 0.01, ***p< 0.001, ****p< 0.0001. BV/TV, bone volume/total volume. Tb. Sp, trabecular separation. Tb. N, trabecular number. Tb. Th, trabecular thickness. BMD, bone mineral density.

The concentrations of serum biochemical indices representing bone metabolism are shown in **Figures 2A, B**. OVX significantly increased the serum levels of CTX-I and OC at all time points compared with Sham rats. The BMDs of OVX rats significantly decreased compared with Sham rats as early as week 3 after surgery. The OVX group experienced significant osteoporotic changes in subchondral bone at week 3 compared with the Sham group (**Figure 2C**), whereas the trabecular parameters of the Sham group showed no significant change. Estrogen supplementation reversed changes in biochemical indices and microstructural parameters of subchondral bone due to OVX (**Figures 2D–H**). TRAP staining demonstrated that more TRAP^+^ osteoclasts were observed in the OVX group than in the Sham group (**Figures 2I, J**). After treatment with estrogen, the number of activated osteoclasts was reduced.

### 3.3 Postoperative changes in cartilage of OVX rats over time

**Figure 3:**
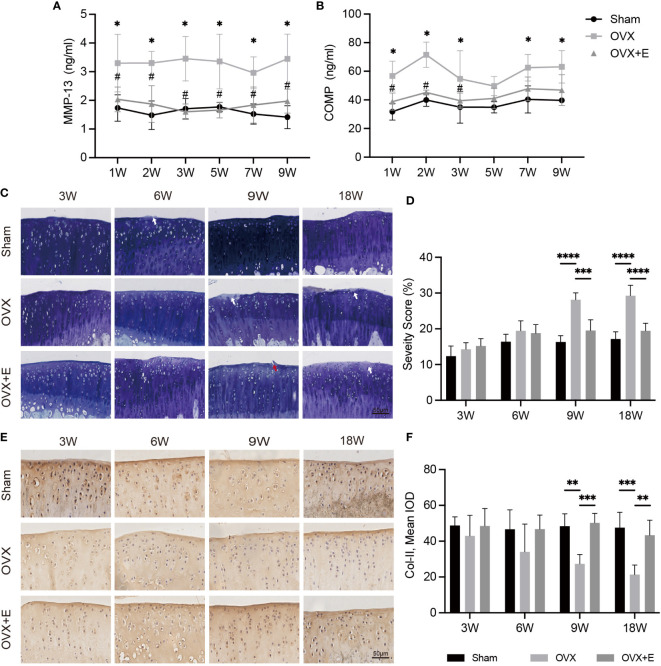
The change in cartilage degeneration after surgery from week 3 to week 18 in Sham, OVX and OVX+E rats. **(A, B)** The serum levels of MMP-13 and COMP (n = 7). *p< 0.05 vs. Sham group. #p< 0.05 vs. OVX group. **(C)** Toluidine blue staining in articular cartilage. The white arrow indicates proteoglycan loss. The red arrow represents the rough surface (n = 7). Scale bar, 50 μm. **(D)** Q uantification of cartilage surface erosion of tibial plateau cartilage. **(E)** Representative images of COL-II expression (n = 7). Scale bar, 50 μm. **(F)** Semiquantitation of COL-II expression. *p< 0.05, **p< 0.01, *p< 0.01, ***p< 0.001, ****p< 0.0001.

The serum levels of MMP-13 and COMP are shown in **Figures 3A, B**. OVX significantly increased the serum levels of MMP-13 and COMP at all time points compared with Sham rats, while the increases were inhibited by estrogen supplementation. Toluidine blue staining was performed to assess articular cartilage damage over time in OVX-OA. The erosion scores increased gradually in OVX rats (**Figure 3C**). The erosion scores were not significantly different between the Sham and OVX groups until week 9 (**Figure 3D**). Immunohistochemical analysis of COL-II in articular cartilage showed a similar trend (**Figures 3E, F**). Estrogen supplementation suppressed degeneration progression, and OVX-induced cartilage degeneration could be repaired at the early stage by estrogen treatment (**Figures 3C–F**).

### 3.4 The relationship between alterations in subchondral bone and cartilage

**Figure 4:**
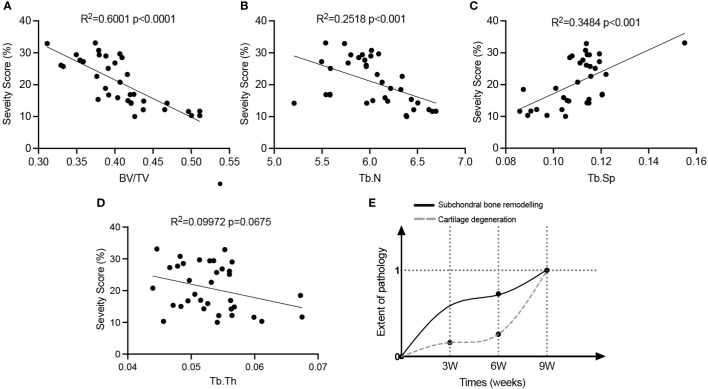
The relationship between subchondral bone alterations and cartilage degeneration in OVX-OA rats. **(A–D)**: Linear regression analysis of the severity of articular cartilage and microarchitectural changes of subchondral bone (n = 35). **(E)** The developed model illustrating pathologies of subchondral bone and cartilage in OVX-OA. BV/TV, bone volume/tissue volume. Tb. N, trabecular number. Tb. Sp, trabecular separation. Tb. Th, trabecular thickness.


Linear regression analysis was conducted to elucidate the relationship between subchondral bone loss and cartilage erosion (**Figures 4A–D**). Except for Tb. Th, the trabecular parameters, such as BV/TV, Tb. Sp and Tb. N, were significantly linearly correlated with cartilage degeneration in the OVX rats. Moreover, a model illustrating the correlation between pathological onset/progression of subchondral bone and cartilage was developed (**Figure 4E**). With the values of BV/TV as the key parameter, the extent of pathological changes in subchondral bone at a certain time was defined as

$$Ext\_Pathol\_bone\;\left(time\right)\;=\frac{Mean\;of\;BV/TV\;of\;OVX\;rats\;\left(time\right)\;-\;Mean\;of\;BV/TV\;of\;Sham\;rats\;\left(time\right)}{Mean\;of\;BV/TV\;of\;OVX\;rats\;\left(9\;w\right)\;-\;Mean\;of\;BV/TV\;of\;Sham\;rats\;\left(9\;w\right)}$$

The time can be 0 w, 3 w, 6 w and 9 w. The definition makes the extent of pathology at week 9 be 1. The extent of pathological changes in cartilage was defined similarly with the values of erosion severity as the key parameter. The model confirmed that the occurrence of significant alterations in subchondral bone preceded the occurrence of significant degeneration in cartilage, and the rate of bone loss was greater than that of cartilage degeneration.

### 3.5 Involvement of TGF-β signaling

**Figure 5:**
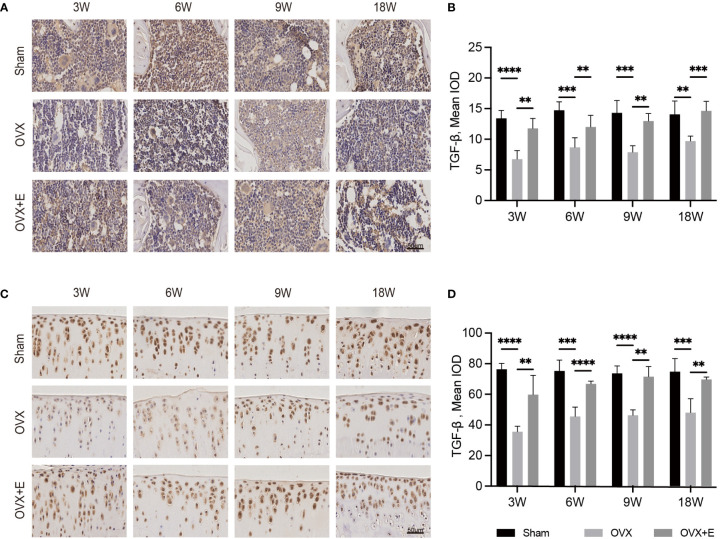
The change in the osteochondral unit involves TGF-β signaling. Immunohistochemical staining of TGF-β in subchondral bone **(A)** and articular cartilage **(C)**. The mean IOD of TGF-β in subchondral bone **(B)** and articular cartilage **(D)** (n = 7). The mean IOD indicates the mean integrated optical density. Scale bar, 50 μm. *p< 0.05, **p< 0.01, ***p< 0.001, ****p< 0.0001.

**Figure 6:**
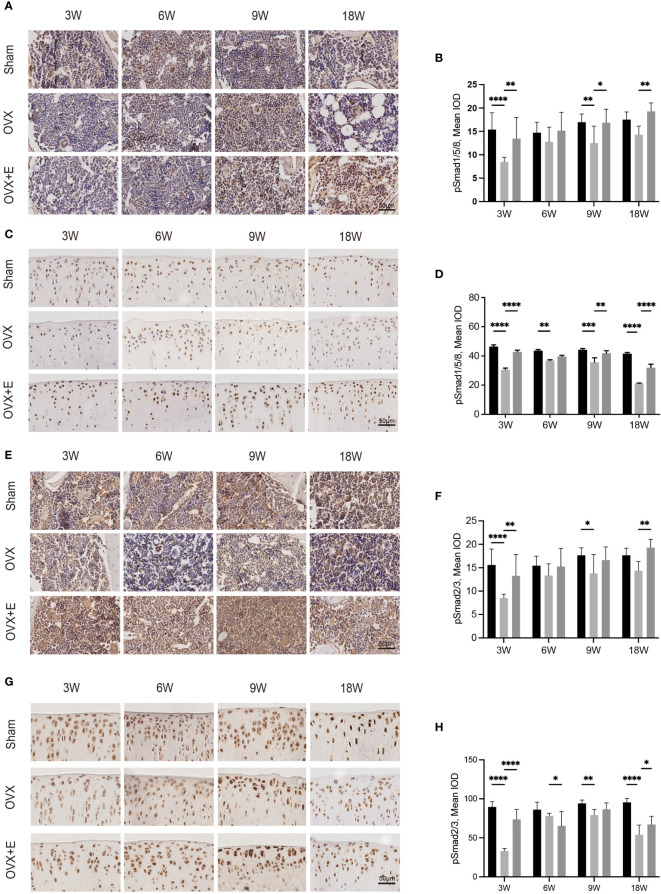
Immunohistochemical staining of pSmad1/5/8 in subchondral bone **(A)** and articular cartilage **(C)**. Immunohistochemical staining of pSmad2/3 in subchondral bone **(E)** and articular cartilage **(G)**. The mean IOD of pSmad1/5/8 in subchondral bone **(B)** and articular cartilage **(D)**. The mean IOD of pSmad2/3 in subchondral bone **(F)** and articular cartilage **(H)** (n = 7). The mean IOD indicates the mean integrated optical density. Scale bar, 50 μm. *p< 0.05, **p< 0.01, ***p< 0.001, ****p< 0.0001.

The expression of TGF-β and pSmads in the osteochondral unit might provide a clue of the mechanism underlying cartilage degeneration in OVX- OA (**Figure 5**, **6**). Immunostaining analysis revealed that the expression of TGF-β and pSmads in both subchondral bone and cartilage dropped significantly at week 3 after OVX compared with Sham rats. Further decreases were not observed after week 3. This effect was attenuated by estrogen treatment (**Figures 5A–D**). The expressions of pSmad2/3 and pSmad1/5/8, the downstream of the TGF-β signaling pathways, were similar to that of TGF-β (**Figures 6A–H**).

## 4 Discussion

The present study demonstrated that early OVX-OA experiences distinct osteochondral alterations compared with PT-OA, implying different roles of subchondral bone in OVX-OA and PT-OA at the early stage. The linear correlation between alterations in subchondral bone and cartilage during OVX-OA development over time suggests the significant role of subchondral bone in OVX-OA.

The effects of subchondral bone on cartilage degeneration under estrogen deficiency deserve intensive investigation. However, the majority of previous studies investigating the role of subchondral bone in OA utilized the PT-OA model (19, 20), which is suitable to simulate OA induced by rapid joint instability. The present study confirmed that the changing rates and degeneration patterns of the osteochondral unit were different in PT-OA and OVX-OA. To investigate the correlation between cartilage and subchondral bone in postmenopausal women, OVX-OA might be the most ideal model. The different pathologies of OVX-OA and PT-OA possibly reflect the different roles of subchondral bone in cartilage degeneration. The rapid and severe cartilage damage in PT-OA can be attributed to the abrupt change in the mechanical environment caused by the surgeries. Probably for this reason, subchondral bone formation increased at week 9 in PT-OA to adapt to the mechanical environment changes. For OVX-OA, the suppressed endogenous estrogen is the initial inducing factor, which directly stimulates bone remodeling and significant bone loss. Comparatively mild cartilage damage was observed at week 9 in OVX-OA, which is consistent with findings in other studies (16). In OVX-OA rats, cartilage degeneration is also possibly directly caused by estrogen. Our previous study proved that cartilage degeneration, at least partly, responds to subchondral bone degradation in OVX-OA by comparing the femur end and tibia end in the same joint, which may best eliminate the direct influence of estrogen on cartilage (10). The distinct osteochondral alterations in OVX-OA give impetus to exploring the evolution law of the osteochondral unit in OVX-OA.

Investigating the alteration evolution of cartilage and subchondral bone over time in OVX-OA helps to better understand which part of the osteochondral unit plays a leading role in early OVX-OA. After OVX, significant increases of the serum levels of MMP-13 and COMP were detected at week 1, which reflected the activated systemic turnover of cartilage due to OVX. Our previous study showed that the serum levels of C-telopeptide of type II collagen (CTX-II) were also significantly increased soon after OVX (9, 21). At the tissue level, subchondral bone is sensitive to estrogen deficiency (22). Significant pathological alteration of subchondral bone, as shown by BMD, microstructure and osteoclast activity, occurred at or possibly before week 3 after OVX. However, statistically notable cartilage erosion and decreased expression of COL-II were not observed until week 9 after OVX. Subchondral bone loss occurred 6 weeks earlier than cartilage degeneration, implying that abnormal subchondral bone remodeling after OVX might contribute to OVX-OA initiation and progression. Analyzing the data about the changes over time showed that cartilage erosion severity is linearly correlated with most subchondral trabecular parameters and bone remodeling biochemical indicators. A proposed model, illustrating the onset and progression of pathology for both subchondral bone and cartilage following estrogen withdrawal, intuitively showed more rapid degradation of subchondral bone compared with cartilage degeneration (**Figure 4**). Both the linear relationship obtained from the curve fitting and the pathological model quantitatively revealed the internal law of action between subchondral bone and cartilage in OVX-OA.

Changes in subchondral bone can affect its ability to dissipate the load and distribute the strain, and thus initiate or accelerate cartilage degeneration (23, 24). The early BMD reduction and microstructural changes in subchondral bone due to OVX may be a deleterious factor for cartilage integrity through disruption of the normal mechanical balance within the osteochondral unit. Moreover, cellular interactions between cartilage chondrocytes and bone cells were reported to play significant roles (25). When cells in subchondral bone are under pathological conditions, the irregular cellular interaction between cartilage and subchondral bone might result in cartilage degeneration (26). In this study, elevated bone remodeling was observed in the subchondral trabecular bone, as reflected by subchondral bone microstructure changes and increased osteoclast activities. The elevated activity of osteoclasts not only results in a separated trabecular network (27), but also accelerates cartilage degeneration. Preosteoclasts can invade the hypertrophic area of cartilage to disturb cartilage matrix remodeling and form a primary ossification center (28, 29). The migration of osteoclast precursors into the cartilage layer enables osteoclasts/precursors to directly interact with chondrocytes. Mature osteoclasts can function as direct regulators of neighboring chondrocytes by secreting various cytokines (30, 31). Estrogen deficiency leads to increased activities of osteoclasts and active bone resorption. Our study did observe an increase in TRAP staining in subchondral bone after OVX.

TGF-β signaling was recognized as one of the important signals in the crosstalk of subchondral bone and cartilage in OVX-OA. TGF-β and its downstream pSmads molecules were all decreased after OVX. Decreased expression of TGF-β in the osteochondral unit was observed in OVX rats, which is consistent with other reports (9, 21). In contrast, increased expression and activity of TGF-β in subchondral bone was found in PT-OA (11, 32). The different expression of TGF-β in OVX-OA and PT-OA partly explains the different alterations of subchondral bone at week 9 in OVX-OA and PT-OA, considering that the activity of TGF-β in bone can promote bone formation and inhibit bone resorption (33). In OVX rats, TGF-β stayed at low levels over time in the cartilage layer compared with Sham rats. There have been considerable studies about the functions of TGF-β in cartilage homeostasis, showing that TGF-β promotes the initial stages of chondrocyte differentiation but represses terminal hypertrophic differentiation (34). After OVX, the decreased TGF-β in the cartilage might have negative effects on maintaining cartilage integrity. We also noticed that abnormally increased TGF-β expression in the subchondral bone in PT-OA can promote cartilage degeneration (35, 36). Therefore, the expression and roles of TGF-β in different types of OA and at the different stages of OA are different. The actual mechanism is complex and demands in-depth investigation.

A limitation of the study needs to be mentioned. No human data are available in this study. The main hypothesis of the present study is that changes of subchondral bone precede cartilage degeneration at the early stage of OVX-OA. Only animal models were used to track the changes of the osteochondral unit over time at the early stage after estrogen withdrawal. It is hard to verify this hypothesis in humans because all the OA patients with records in our hospital underwent surgeries and mostly represented the late stage of OA. Better design of a prospective study which includes OA participants with different ages and different pathogenies is warranted in the future.

In summary, OVX-OA demonstrated distinct osteochondral alterations from PT-OA, and the pathological changes were time-dependent. Subchondral bone changes occur earlier than cartilage changes and may play a vital role in the OVX-OA process. The osteochondral changes were correlated with aberrant expression of TGF-β and its downstreaming molecules in the osteochondral unit. These findings exhibited the unique features of OVX-OA, especially the role of subchondral bone, and provided more justification for targeting subchondral bone treatment as an early intervention strategy for postmenopausal OA.

## Data availability statement

The raw data supporting the conclusions of this article will be made available by the authors, without undue reservation.

## Ethics statement

The animal study was reviewed and approved by Animal Ethics Committee of Peking University.

## Author contributions

ZY, QT, and HL were involved in all aspects of the project from initial conception to writing of the manuscript. ZZ, GN, SL, WL, and CS were engaged in the methodology and data collection. All authors contributed to the article and approved the submitted version.
